# Proteomic subtyping highlights tumor heterogeneity of human HCC

**DOI:** 10.1007/s00428-025-04260-w

**Published:** 2025-10-03

**Authors:** Thomas Ritz, Jovan Tanevski, Jana Baues, Sven H. Loosen, Tom Luedde, Ulf Neumann, Peter Boor, Peter Schirmacher, Julio Saez-Rodriguez, Thomas Longerich

**Affiliations:** 1 Institute of Pathology, University Hospital Heidelberg, Im Neuenheimer Feld 224, 69120 Heidelberg, Germany; 2 Institute for Computational Biomedicine, Heidelberg University & Heidelberg University Hospital, Faculty of Medicine, Heidelberg, Germany; 3 Institute of Pathology, Uniklinik RWTH Aachen, Aachen, Germany; 4 Department of Gastroenterology, Hepatology and Infectious Diseases, University Hospital Dusseldorf, Düsseldorf, Germany; 5 Department of General-, Visceral- and Transplantation Surgery, University Hospital Essen, Essen, Germany

**Keywords:** HCC, Multiplexed immunofluorescence, Intratumoral heterogeneity, Multispectral imaging

## Abstract

Hepatocellular carcinoma (HCC) has a poor prognosis. While molecular profiling has identified subclasses with potentially druggable pathways, implementation in routine diagnostics remains challenging. Although immunohistology may aid HCC classification, multiplexed protein-based approaches have not yet been established. Proteomic heterogeneity in HCC tissue also remains poorly understood. Tissue microarrays from 58 HCC patients were analyzed using a multispectral imaging platform, enabling the detection of multiple protein biomarkers on a single tissue slide. A machine learning-based algorithm facilitated single-cell expression analysis, clustering, and spatial distribution assessment. A 4-plex immunofluorescence marker panel was designed and applied to interrogate altered signaling pathways in HCC. Unsupervised analysis revealed four factors corresponding to three HCC clusters defined by the overexpression patterns of p-S6/CRP (Cluster A), glutamine synthetase (Cluster B), and EpCam (Cluster C). Single-cell resolution uncovered substantial intratumoral heterogeneity. Only one third of HCCs showed a ≥ 0.95 purity of tumor cells in the predominant cluster. Clinically, Cluster C was associated with reduced median overall survival, while the other clinico-pathological features were not significantly different between the clusters. A protein-based subclassification of human HCC was established, characterized by three distinct subclasses (inflammation, beta-catenin/WNT signaling, progenitor-like) that align with known molecular categories. Cases with dominant progenitor features tended to have a shorter survival probability. The intratumoral heterogeneity observed in most cases may promote therapy resistance and underscores the need for precise molecular stratification to improve treatment outcomes.

**Supplementary Information:**

The online version contains supplementary material available at 10.1007/s00428-025-04260-w.

## Introduction

Primary liver cancer, with hepatocellular carcinoma (HCC) being the most prevalent type, is a major global health burden and ranks as the third leading cause of cancer-related deaths worldwide [1]. HCC is a highly heterogeneous disease, exhibiting significant morphological and molecular diversity. High-throughput sequencing studies have identified distinct molecular subclasses of HCC, some of which display a clinically aggressive behavior with early recurrence and poor survival probability [2, 3]. The individual subclasses are defined by genomic alterations and altered signaling pathways, such as the Wnt/β-catenin and AKT/mTOR pathways. Boyault’s classification categorizes HCC into six subgroups (G1–G6) with distinct molecular characteristics. For instance, the G5 and G6 subgroups are associated with *catenin beta 1* (CTNNB1) gene mutations leading to Wnt/β-catenin-pathway activation, while G2-G3 subgroups often show *tumor protein P53* (TP53) gene mutations [4].

However, despite the availability of comprehensive molecular profiling, the implementation of these findings in routine diagnostics is limited by the complexity of sequencing-based approaches. Immunohistochemistry (IHC) represents a solid, easy, widely available, and low-cost approach to the interrogation of pathway components based on the expression level of surrogate protein markers. Calderaro et al. promoted this concept by proposing specific immunohistochemical markers that reflect the subclasses of the Boyault classification [5]. In the current approach, we selected four markers representing key axes of HCC diversity as described in transcriptome-based classifications. Glutamine synthetase (GS) is used as a marker for Wnt-signaling activation, while C-reactive protein (CRP) and phospho-S6 ribosomal protein (p-S6) indicate activation of inflammatory and mTOR-related pathways, respectively. Epithelial cell adhesion molecule (EpCAM), a marker of liver progenitor cells, was associated with aggressive tumor behavior and poor prognosis [6].

Our study aimed to independently validate the utility of immunohistochemistry for subclassification of HCC by employing a novel multiplexed immunohistochemical panel combined with multispectral imaging. This innovative approach facilitates spatially resolved, single-cell level analysis of protein markers, overcoming the limitations of traditional bulk or single-marker analyses. Integrating this methodology into tissue-based research, we bridge the gap between advanced molecular insights and routine diagnostic workflows. Ultimately, this strategy not only offers a robust tool for subclassifying HCC but also has the potential to enhance therapeutic decision-making by linking molecular tumor characteristics to treatment responses in a clinically practical manner.

## Material and methods

### Patient cohort and tissue microarrays (TMA)

Formalin-fixed and paraffin-embedded (FFPE) tissue from resection specimens obtained from 58 patients with HCC between 2010 and 2016 were retrieved from the archive of the Institute of Pathology, University Hospital, RWTH Aachen. Only patients who underwent resection or liver transplantation were included; biopsy specimens were not considered. The study was approved by the local ethics committee (Nr. EK 122–16). Tissue slides were re-evaluated by two experienced pathologists (TR, TL) with special expertise in hepatopathology to confirm the diagnosis of HCC [7]. After microscopy-based selection of informative areas, each two cores of tumor and non-tumor tissue (1.0 mm diameter) were retrieved from each donor block and embedded in a new paraffin recipient block using a microarrayer (Beecher Instruments, Silver Spring, MD, USA). Non-neoplastic liver tissue was included in the TMA design to serve as internal control for staining specificity and quality assurance in the workflow. Cohort characteristics are listed in Table 1.

**Table 1:** Patient characteristics

Parameter	Study cohort
**Cancer patients**	*n* = 58
Gender [%]:	
Male–female	74–26
Risk factors [%]	
HBV	19
HCV	12
Alcohol	7
Metabolic	9
Typ 2 diabetes, past HBV	2
Typ 2 diabetes, alcohol	5
PBC	3
HBV/HCV	2
Unknown	41
Cirrhosis [%]	
No	50
Yes	50
Histological grading [%]	
G1	14
G2	41
G3	43
Specific subtype	2
UICC [%]	
UICC I	31
UICC II	41
UICC III	24
UICC IV	4
Lymphangiosis carcinomatosa [%]	
No	91
Yes	9
Hemangiosis carcinomatosa [%]	
No	57
Yes	43

*HBV* hepatitis B virus, *HCV* hepatitis C virus, *PBC* primary biliary cholangitis, *UICC* Union Internationale Contre le Cancer

### Chromogenic stainings

Chromogenic immunohistochemistry (cIHC) was performed on 4-µm tissue sections from the TMA using an automated immunostainer (Ventana BenchMark Ultra, Ventana Medical Systems, Tucson, AZ, USA), as described before [8]. Briefly, a 32-min pre-treatment was followed by a 24-min incubation with the specific antibodies for C-reactive protein (CRP; clone Y284, Abcam, Cambridge, MA, USA, 1/2000), epithelial adhesion molecule (EpCam; clone Ber-EP4, Agilent, Santa Clara, CA, USA, 1/400), glutamine synthetase (GS; clone GS-6, Merck, Darmstadt, Germany, 1/500), and phosphorylated ribosomal S6 kinase (pS6; clone 2211, Cell Signalling Technology, Danvers, MA, USA, 1/200) at 36 °C. Subsequently, an 8-min incubation with OptiView Universal Linker (Ventana) was performed. Visualization was achieved by incubation with DAB for 8-min (OptiView HRP Multimer, Ventana).

### Panel design and multiplexed immunofluorescence (mIF)

Immunofluorescence was performed using 3-µm tissue sections from the TMA blocks and the Opal multiplex Manual Detection Kit (NEL811001KT, Akoya Biosciences, Marlborough, MA USA). The antibody panel, which was based on the subclassifying antibodies proposed by Calderaro et al. [5], consisted of CRP, EpCam, GS, and p-S6 and was optimized according to the manufacturer’s instructions. In brief, after epitope retrieval (PT Link Pre-Treatment Module, Agilent, Santa Clara, CA, United States) and endogenous peroxidase blocking (EnVision™ FLEX Peroxidase-Blocking Reagent, Agilent, Santa Clara, CA, United States), the respective primary antibody was incubated for 30 min before an HRP-tagged, host-specific secondary antibody was applied for 20 min (ImmPRESS® HRP Polymer Detection Kit, Vector Laboratories, Newark, CA, USA), both at room temperature. For signal amplification, a 10 min incubation was performed using different tyramide signal amplification dyes (Opal 690, Opal 540, Opal 570 and Opal 520, Akoya Biosciences, Marlborough, MA, USA) diluted 1:100. After each staining cycle, a microwave oven stripping procedure was applied to ensure removal of antibody complexes not covalently bound to their cognate targets, thus facilitating detection of the next antigen without increasing the risk of cross-reactivity. In detail, slides were placed vertically in slide processing jars filled with antigen retrieval buffer and subjected to microwave treatment. The microwave was set to full power until the buffer reached boiling (100 °C), as determined by a temperature probe. Following this, slides were microwaved at 20% power for 15 min to maintain a constant boil. Buffer levels were monitored to ensure they remained above the tissue level to prevent drying. The dilutions of the primary antibodies were adjusted and validated to allow proper detection using the tyramide amplification system (CRP 1/15000, Opal 570; EpCam: 1/3200, Opal 540; GS: 1/1000, Opal 690; p-S6: 1/3200, Opal 520). For nuclear staining, slides were incubated with 4′,6-diamidino-2-phenylindole (DAPI) for 5 min at room temperature. After validating the staining characteristics of the individual monoplex stainings, optimization of antibody dilutions, and determination of the optimal staining sequence, TMA slides were sequentially stained with the four individual antibodies. Slides were mounted using Vectashield® Mounting Medium (VectorLaboratories, Newark, CA, USA). To exclude that the sequential incubation of the different antibodies resulted in an artificial staining pattern, mIF and cIHC staining pattern were compared at the single antibody level first. The dilution of each antibody used for tyramide signal amplification-based mIF was adjusted to achieve both a comparable intensity and pattern of staining. This optimization process involved fine-tuning of antibody concentrations and incubation times. An exemplary comparison of both staining techniques is shown in Supp. Fig. 1.

### Multispectral imaging (MSI)

**Fig. 1:**
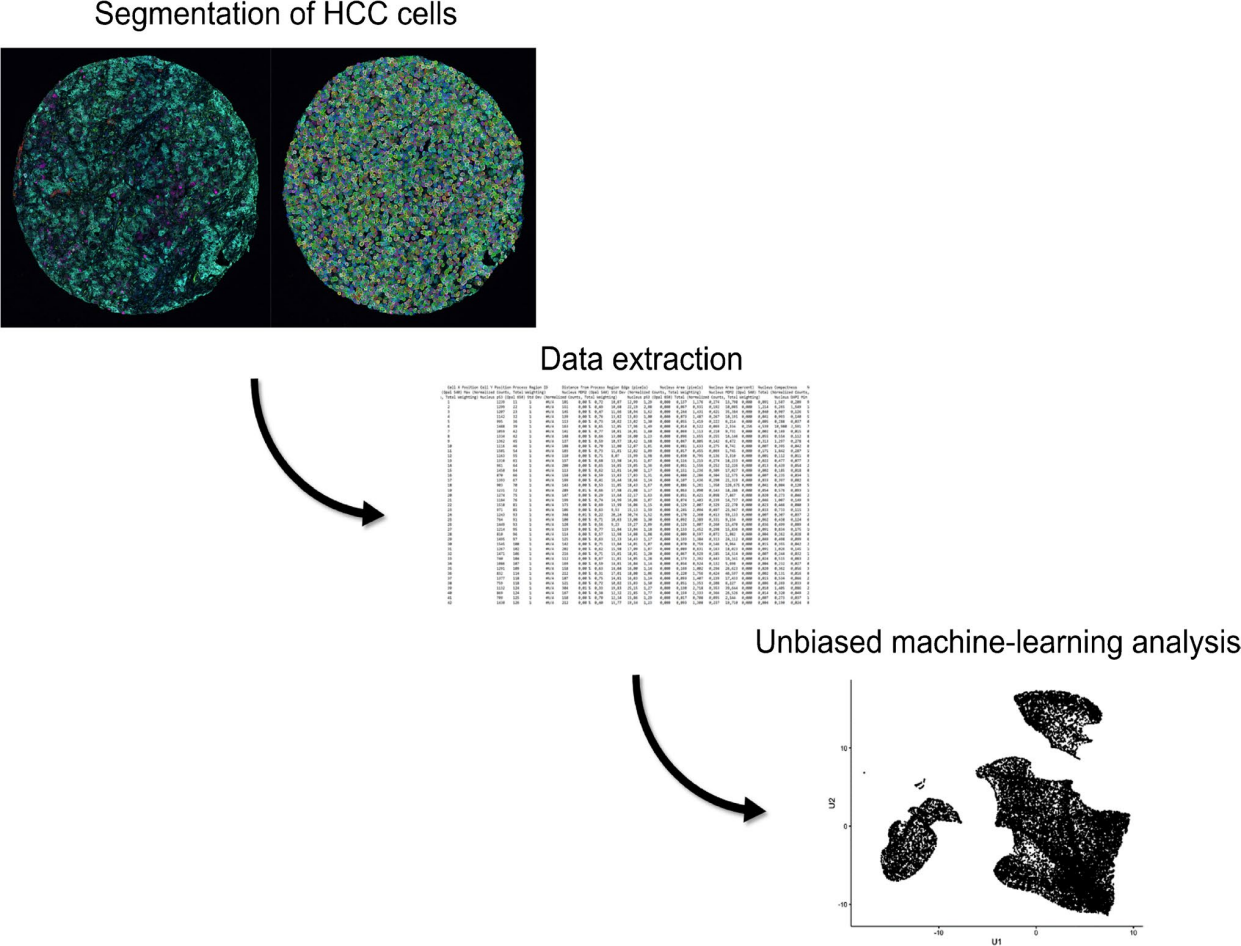
Workflow of data acquisition and analysis. Algorithm-based and individually adjusted segmentation of HCC tumor cells, followed by data extraction of a single-cell-based protein expression profile for the markers EpCam, CRP, pS6, and GS. Expression profiles were then analyzed in an unbiased, machine learning-based approach

MSI was performed using the Vectra 3 quantitative pathology imaging system (Akoya Biosciences, Marlborough, MA USA). To ensure accurate unmixing of the partly overlapping fluorophore spectra and to extract tissue autofluorescence, spectral libraries were generated for each fluorophore as well as the normal liver tissue. After whole slide scanning of the TMA slides at low magnification (×10) and assignment of the TMA-tissue cores, MSI was performed at higher magnification (×20) using the five fluorescence filters Cyanine3 (Cy3), Cyanine5 (Cy5), DAPI, fluorescein isothiocyanate (FITC), and Texas Red. Multispectral images were analyzed using the InForm software (Akoya Biosciences, Marlborough, MA USA). After training a machine learning-based algorithm for tissue and cell segmentation, the quality of segmentation was validated for every core and if necessary adjusted to ensure correct data analysis. Expression raw data were exported for further analysis (Fig. 1).

### Data pre-processing

After cell segmentation, the expression of each marker for each HCC cell was represented by the normalized IF counts with total weighting. For each marker, expressions were considered outliers if they were outside of the range [Q1—1.5·IQR, Q3 + 1.5·IQR], where Q1 and Q3 are the lower and upper quartiles of expression of the marker across all cells, and IQR is the interquartile range. Outlier values were winsorized, i.e., set to the limits of the range Q1—1.5·IQR or Q3 + 1.5·IQR, respectively, to retain the total number of cells. Next, to remove the effect of marker abundance during downstream processing, the expressions levels were quantile and then rank normalized per marker.

### Dimensionality reduction and clustering

For visualization purposes, the normalized dataset was reduced to two dimensions using UMAP (R package uwot 0.1.10) with 100 approximate nearest neighbors and a minimum distance of 0.2 between points in the low dimensional space [9]. Factorization and clustering hepatocyte expression patterns were identified by consensus non-negative matrix factorization minimizing a least squares-based cost function enforcing sparsity on the coefficient matrix (SNMF/R, R package NMF 0.23.0) [10]. Regularizing the coefficient matrix contributes to cleaner cluster assignment of the hepatocytes to an expression pattern as represented by the columns of the basis matrix. The rank of the basis matrix was selected such that both the consensus cophenetic correlation coefficient and the sparseness of the coefficient matrix are maximized. Consensus was established over ten independent runs of the SNMF/R algorithm. The factor analysis was performed on cells from all available cores per patient. Due to specimen loss, only a single core was available for evaluation in 16 patients. HCC cells were assigned to a cluster based on the maximum coefficient for each cell in the resulting coefficient matrix. The quality of the clustering was evaluated by the silhouette score given the quantile normalized expression values for each HCC cell. The silhouette score quantifies how well each cell fits within its assigned cluster compared to neighboring clusters, calculated as (*b*—*a*)/max(*a*, *b*), where *a* is the mean distance to other cells within the same cluster, and *b* is the mean distance to cells in the nearest neighboring cluster. Silhouette scores range from −1 to 1, where values near 1 indicate that the cell is well-matched to its own cluster and poorly matched to neighboring clusters, and negative values suggest that the cell may have been assigned to the wrong cluster. To improve the confidence of downstream analyses, cells with zero or negative silhouette scores were removed, leaving only representative HCC cells with clear cluster membership. After this cluster assignment, a purity score was calculated for each core as the fraction of hepatocytes belonging to the most frequent cluster in the core. A purity score of 1 indicates a homogeneous core, where all tumor cells were assigned the same cluster label, while a score of 0.33 represents the most heterogeneous core. Figure 1 summarizes the methodological approach.

### Differential marker expression profiles

The differential expression profiles of each were calculated as the one-vs-all difference in mean expression for each marker after removing cells with negative silhouette scores. The significance of the difference in means was quantified by false discovery rate (FDR) corrected *t*-tests. The differential marker expression analysis was performed in R.

### Statistical analyses

All other statistical analyses were performed using SPSS version 23 (SPSS Inc., Chicago, IL, USA). Cross-tabulations were generated to analyze descriptive data. Pearson’s chi-square test was applied to compare binary and nominal variables. A *p*-value of < 0.05 was considered statistically significant. The survival probability of the different clusters was further studied with Kaplan–Meier curves. Curves were compared using log-rank test. Patients were censored at the time of lost follow-up (last contact to physician) or death.

## Results

### Single-cell protein expression profiling identifies three different tumor clusters

**Fig. 2:**
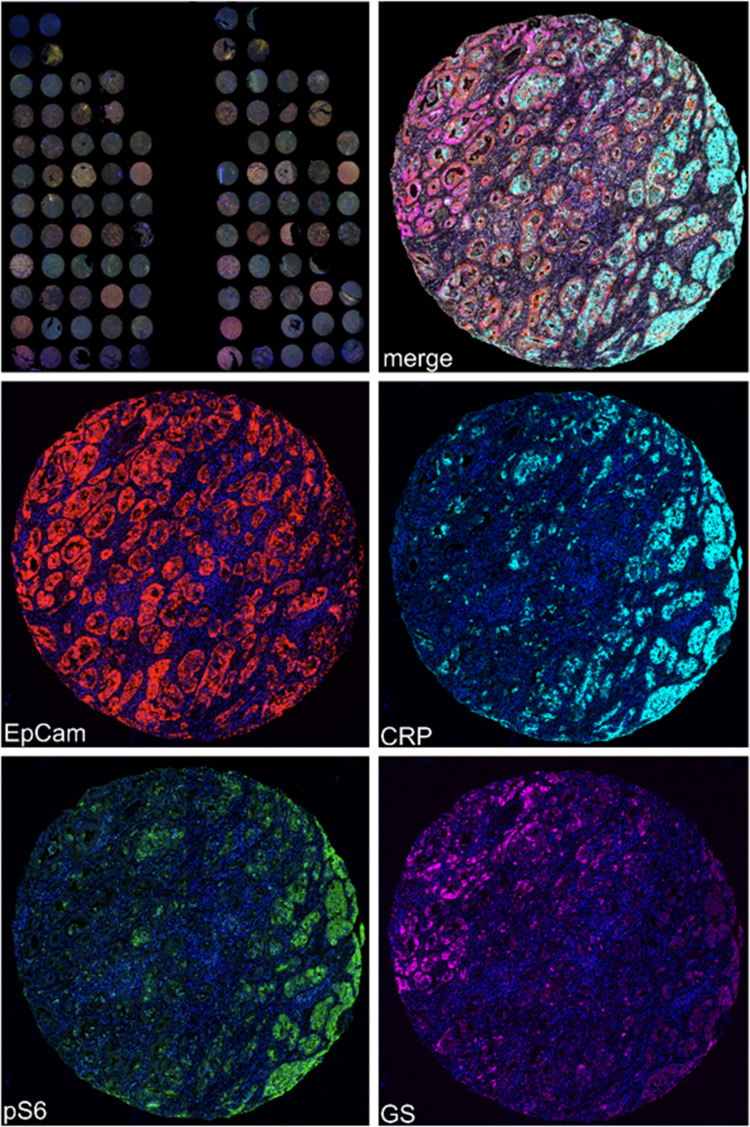
Multispectral images and spectral unmixing. Four-color mIF of human HCC. Multispectral image of a whole slide scan and a single HCC core after spectral unmixing of a four-color mIF. Monoplex stainings for EpCam (Opal 650, red), CRP (Opal 540, light blue), phospho-S6 (Opal 570, green) and GS (Opal 690, magenta) after unmixing

**Fig. 3:**
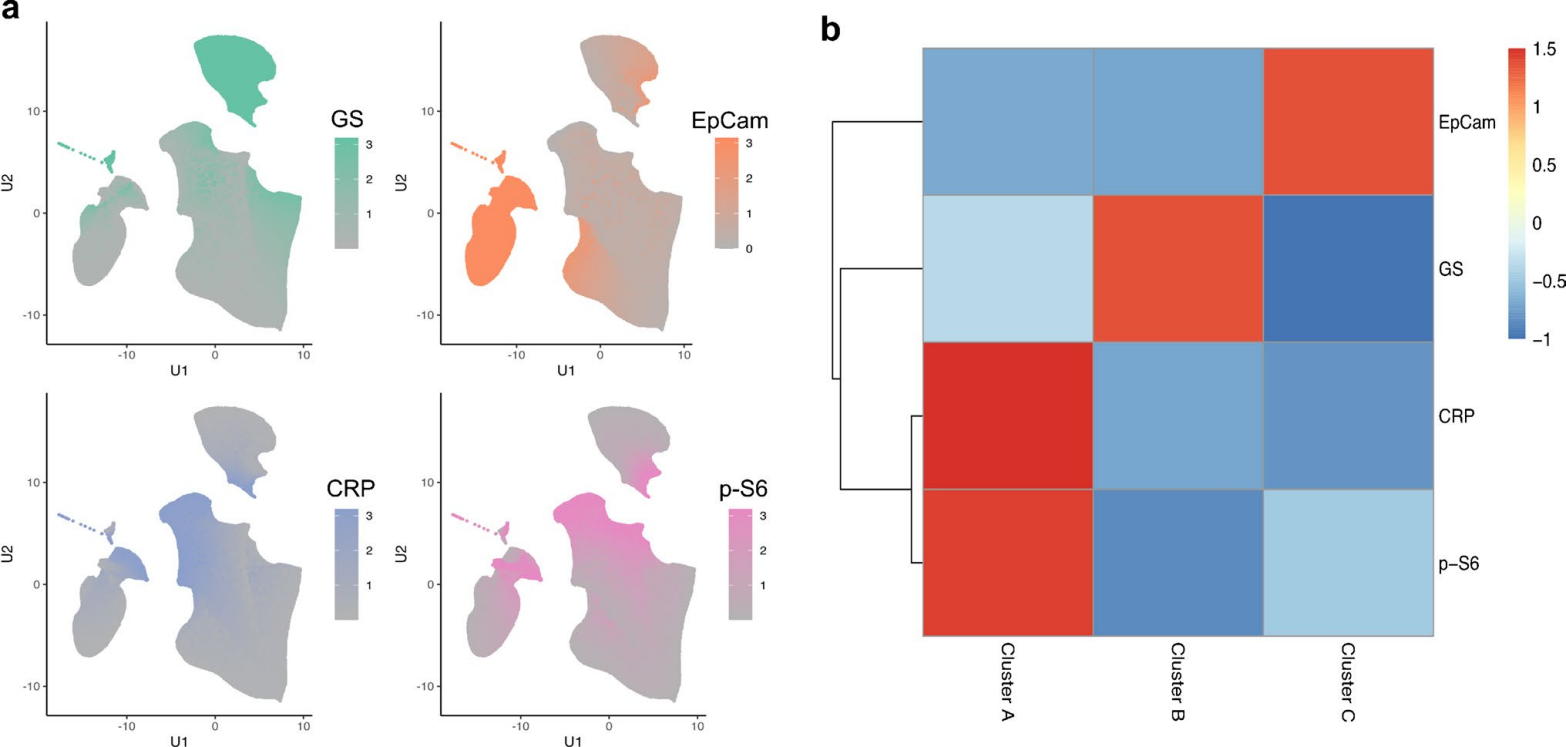
Clustering based on biomarker expression. **a** The data for Uniform Manifold Approximation and Projection (UMAP) for dimension reduction was obtained by multispectral imaging measuring TMA cores from 58 patients. **b** Clustering by factor analysis identified three clusters. Cluster A showed high p-S6 and CRP expression, Cluster B was characterized by diffuse GS upregulation, and Cluster C revealed overexpression of the progenitor cell marker EpCam

Hepatocarcinogenesis is associated with alterations in protein expression due to tumor cell mutations and altered cellular signaling. To assess the expression of marker proteins on a single tumor cell basis, multiplex immunostaining using antibodies against CRP, EpCam, GS, and phospho-S6 was performed (Fig. 2). Stromal cells and immune cells were excluded from further analysis by step-wise tissue and cell segmentation. After cell segmentation, expression data of 223,846 HCC cells from 58 patients were extracted. The number of cells per TMA core ranged from 84 to 15,337. To improve data consistency, only cells with positive silhouette scores (Supp. Tab. 1) were used for further analysis. Thus, a median number of 2699 tumor cells (range: 73 to 14,423) per core was included. Subsequent analysis of the multidimensional dataset revealed three clusters: Cluster A was characterized by expression of p-S6 and CRP. Cluster B included the cases with diffuse expression of GS, while Cluster C contained the EpCam-positive HCC cells (Fig. 3). The total number of cells assigned to each cluster across all 58 samples was 57,755 in Cluster A, 66,385 in Cluster B, and 65,525 in Cluster C.

### The cluster distribution reveals tumor heterogeneity

Next, we analyzed the distribution of the respective clusters across the different tumor samples in more detail. Cluster A cells were dominant in 24 tumors (41%), Cluster B cells were the main constituent in 21 tumors (36%), and Cluster C characterized the main cell type in the remaining 13 tumors (22%). Noteworthy, the main cluster differed between the two cores of the HCC sample in 16 cases, suggesting intratumoral heterogeneity (Suppl. Table 1).

**Fig. 4:**
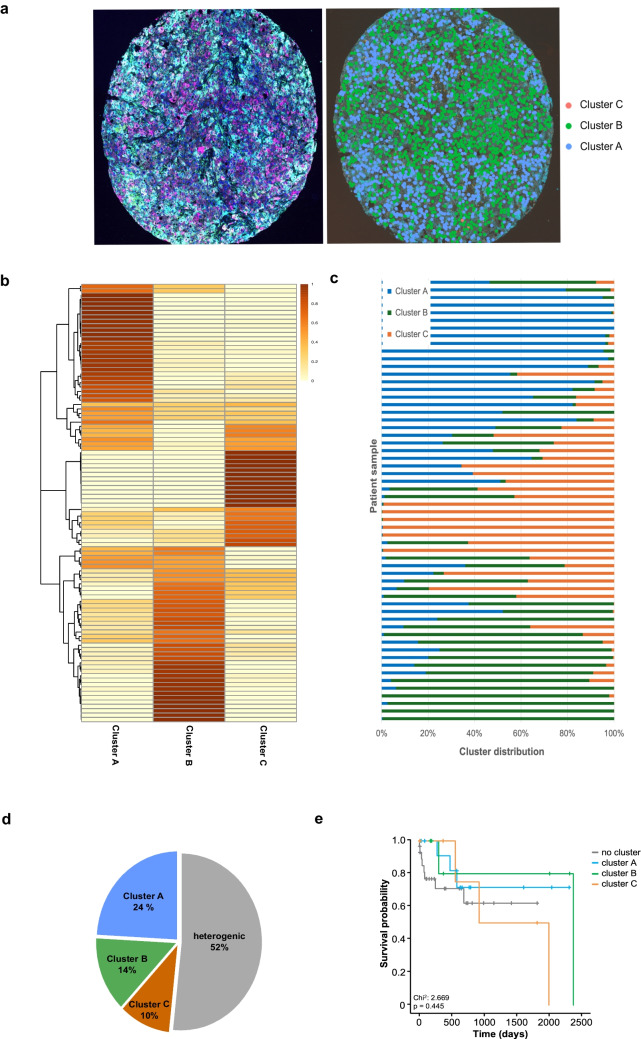
Cluster distribution and survival probability. **a** Multispectral image (left) and overlay of single cell cluster information (right). **b** Hierarchical clustering of HCC samples by purity. **c** Percentage of cells per clusters for each patient. **d** Cluster distribution of the entire cohort at a cluster purity of ≥ 0.80. **e** Survival probability based on cluster distribution

To address this observation in more detail, the data of all HCC cells were considered for each case, and a purity score was calculated for each dominant cluster (Suppl. Table 1). Intratumoral heterogeneity was a prevalent feature in this cohort of human HCC. Only 33% (*n* = 19/58) of HCC cases revealed a purity score ≥ 0.95. In line with this finding, the purity score was significantly higher in cases with an identical compared to a divergent main cluster (mean: 0.86 ± 0.16 vs. 0.55 ± 0.09, *p* < 0.0001). The median purity score was 0.80 (range: 0.34 to 1.00). This cut-off was used to discriminate HCCs with low and high purity. Importantly, the number of HCC cells available for evaluation did not significantly differ between cases with low and high purity (3242 ± 537 vs. 3300 ± 424, *p* = 0.65), respectively. Next, a reverse analysis transducing the single-cell cluster information back to the original multispectral images was performed. This approach facilitated the visualization of the cluster information at single-cell level in each tumor sample, with about half of tumors being clearly assigned to one of the three clusters (e.g., purity score > 0.8), while the others (*n* = 30/58) revealed a higher degree of heterogeneity at the cellular level (Fig. 4a–c). Importantly, even in tumors with a high purity, there were HCC cells belonging to another molecular cluster (Fig. 4c, Supp. Tab. 1).

### Association between clusters and clinical characteristics

To cope with the high proportion of cases with intratumoral heterogeneity, we re-categorized the HCC cases based on the purity of the dominant cluster; 24% of cases (*n* = 14) showed a purity of Cluster A cells > 0.80, while cluster B was dominant in 14% of HCCs (*n* = 8), and 10% of tumors (*n* = 6) were predominated by Cluster C cells. The remaining 52% of HCCs (*n* = 30) with lower purity were considered not belonging to a defined cluster (Fig. 4d).

To get a deeper insight into the biological relevance of the cluster purity in human HCC, the available clinical and histopathological data were compared between these subgroups. There were no statistically significant differences regarding the underlying tumor etiologies, the prevalence of liver cirrhosis, HCC grading, tumor burden, and UICC stage between the clusters (each *p* > 0.05). Survival analysis revealed a reduced median overall survival for HCC patients assigned to Cluster C compared to Cluster B (922 vs. 2376 days, *p* = 0.445), whereas the median survival was not reached for HCCs of Cluster A and the “no cluster” cases (Fig. 4e).

In summary, proteomic HCC subclasses were similar regarding most clinical and pathological characteristics, but HCCs with a high purity of Cluster C cells exhibit a more aggressive clinical course.

## Conclusion

The expanding array of therapeutic options for HCC patients is limited by tumor heterogeneity, a factor contributing to treatment failures due to selection of resistant clones [11, 12]. Therapeutic stratification of patients may be used to address this challenge. Various methodologies, including gene signature-based, metabolic, and immunological classifications, have been explored for HCC patients [13–16]. Comprehensive genomic and transcriptomic studies have identified various molecular subclasses of HCC, each with distinct genetic alterations and clinical outcomes. These findings have been pivotal in revealing the complexity of HCC and identifying potential therapeutic targets. Despite these advances, integrating molecular profiling into routine clinical practice remains challenging due to the high costs and technical complexities involved. Currently, data on protein-based subclassification of HCC are limited but hold great promise for enhancing diagnostic precision and personalized treatment strategies. Given that biopsies are nowadays recommended for many HCC patients for either diagnostic or therapeutic decision-making, incorporating a protein-based classification system into biopsy analysis could improve patient stratification and treatment planning [17]. The emerging field of multiplexed Immunofluorescence (mIF) platforms, initially designed to explore the tumor microenvironment in the context of immuno-oncology [18, 19], inspired our study to address this gap by demonstrating that mIF can effectively subclassify HCC based on easily obtainable protein expression data. Our protein-based subclassification of HCC reveals that only about half of human HCCs can be assigned to a rather homogeneous proteomic cluster that align with the molecular classifications described in the literature [4, 5, 13, 14, 20]. Specifically, Cluster A, characterized by p-S6 and CRP overexpression, mirrors features of Boyault classes G3/G4 and Hoshida’s S1 subtypes, which are characterized by high proliferative activity and inflammatory features. The TCGA data further support the relevance of these pathways, particularly in relation to mTOR pathway activation and TP53 mutations, both of which are frequently associated with inflammation [14]. Cluster B, characterized by β-catenin pathway activation and glutamine synthetase (GS) overexpression, aligns with the WNT-activated subclasses of the Boyault’s (G5/G6) and the S3 subtype of Hoshida’s classification. Cluster C, characterized by EpCAM overexpression, represents the progenitor-like subtypes from previous studies, including Hoshida’s S2 and the G1 subclass described by Boyault. As expected from previous studies [21, 22], HCCs belonging to Cluster C may exhibit a more aggressive biological phenotype, reflected in this cohort by a reduced survival compared to Cluster B HCCs. In contrast, tumor grade, tumor burden, UICC stage, and the presence of cirrhosis did not significantly differ between the clusters, supporting an added value of our molecular stratifying approach to classical histopathological HCC classification.

Another significant finding of our study is the high degree of intratumoral heterogeneity. Divergent dominant clusters were detected between tumor cores in 16 cases, and only one-third of HCCs displayed a purity score ≥ 0.95. Despite being predominantly associated with a specific cluster, the co-existence of cells belonging to different clusters within a single HCC nodule in many patients suggests that the proposed HCC subclasses may not be as stable as expected, but instead may be prone to change, in particular, in response to the selection pressure exerted by specific (systemic) treatments. As a consequence, therapy resistance may evolve. Even more important, our observation underscores that intratumoral heterogeneity is a pervasive feature of HCC, thus highlighting the enormous challenges in assigning the optimal therapy to an individual HCC patient. A similar high proportion of intratumoral heterogeneity was also observed in the PLANet study using a multi-sector, multi-omics approach to characterize the complex genomic and transcriptomic landscape of single HCC nodules [22].

Although comprehensive genomic and transcriptomic assays offer deep insights into HCC’s molecular landscape, they require complex, resource-intensive technologies often unavailable in routine clinical practice. Multiplexed immunofluorescence (mIF) and multispectral imaging directly visualize protein expression in tissue samples, enabling easier use in standard pathology labs without high-throughput sequencing. Capturing cellular diversity within an HCC nodule at the protein level is a key advantage of our immunohistology-based subclassification approach. It detects intratumoral heterogeneity with a fluorescence microscope, revealing distinct tumor cell subpopulations often missed by bulk molecular analyses.

The implementation of a protein-based classification system for HCC holds significant potential for future research and clinical practice. One promising direction is the secondary analysis of existing study cohorts. On one hand, this may validate our findings across larger and more diverse patient populations, further refining the protein-based subtypes and their clinical relevance. Additionally, integrating protein-based classification into clinical study designs could improve patient stratification, enabling tailored therapies for specific subcohorts, potentially enhancing trial outcomes and accelerating subgroup-targeted therapy development. Our study provides a significant leap in the molecular subclassification of HCC by introducing a protein-based methodology that not only aligns with transcriptomic subtypes but also captures intratumoral heterogeneity in unprecedented detail. The ability to visualize intratumoral heterogeneity at single-cell resolution underscores the importance of spatial proteomics for future precision oncology approaches in liver cancer.

However, this study has some limitations. The use of tissue microarrays (TMAs) restricts analysis to small tumor areas and may not fully reflect the spatial complexity of entire tumors. Furthermore, the retrospective design and limited cohort size reduce the statistical power for clinical correlations. Despite these limitations, our findings demonstrate that mIF-based protein profiling is a powerful tool to classify HCC and uncover clinically meaningful heterogeneity, supporting its integration into future diagnostic and translational research.

## Supplementary Information

Below is the link to the electronic supplementary material.

ESM 1 Supplementary Material 1 (DOCX 2.44 MB)

ESM 2 Supplementary Material 1 (DOCX 36.7 KB)

## Data Availability

The datasets generated during and/or analyzed during the current study are available from the corresponding author on reasonable request.

## Abbreviations

HCC: Hepatocellular carcinoma
p-S6: Phospho-S6 ribosomal protein
CRP: C-reactive protein
GS: Glutamine synthetase
EpCam: Epithelial cell adhesion molecule
AKT/mTOR: Protein kinase B/mechanistic target of rapamycin
CTNNB1: Catenin beta 1
FFPE: Formalin-fixed and paraffin-embedded
TMA: Tissue micro array
cIHC: Chromogenic immunohistochemistry
MSI: Multispectral imaging
DAPI: 4′,6-Diamidino-2-phenylindole
FITC: Fluorescein isothiocyanate
Cy3: Cyanine3
Cy5: Cyanine5
UMAP: Uniform Manifold Approximation and Projection for dimension reduction
FDR: False discovery rate
mIF: Multiplexed immunofluorescence
TCGA: The Cancer Genome Atlas
TP53: Tumor protein P53
CK19: Cytokeratin19
