# Supplementary material for: Proteomic subtyping highlights tumor heterogeneity of human HCC
Source: Virchows Arch. 2025 Oct 3;487(5):959–69. doi: 10.1007/s00428-025-04260-w (PMC12647349; doi:10.1007/s00428-025-04260-w)
Supplement: Supplementary file 1 — Supplementary Material 1 (DOCX 2.44 MB) [file 428_2025_4260_MOESM1_ESM.docx]

**Supplementary Information**

**Fig. 1**

**
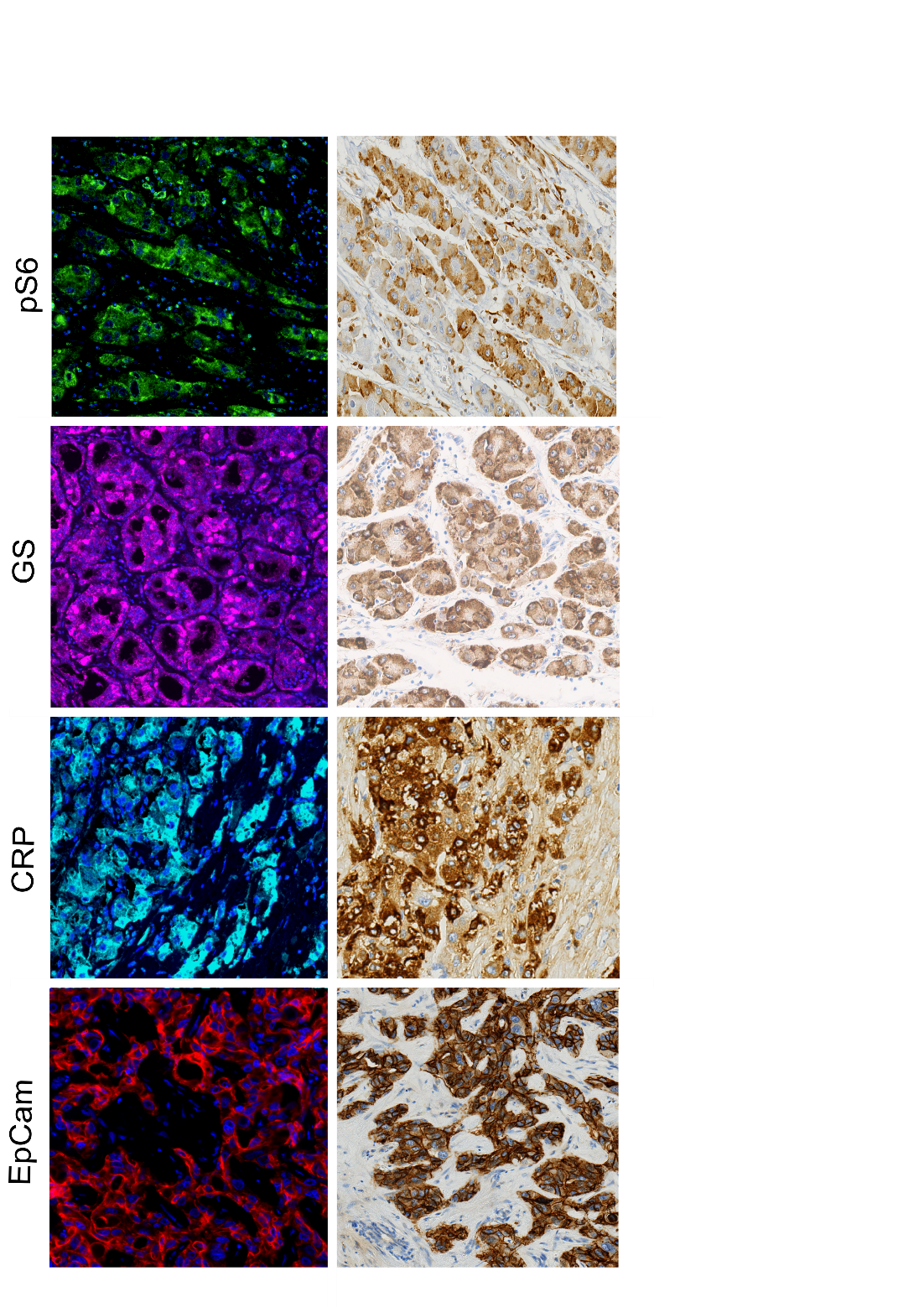
**

**Fig. 1 Multiplexed Immunofluorescence (mIF) vs. chromogenic Immunohistochemistry (cIHC)**

Multiplexed Immunofluorescence staining characteristics are comparable to conventional chromogenic Immunohistochemistry
