# Supplementary material for: Proteomic subtyping highlights tumor heterogeneity of human HCC
Source: Virchows Arch. 2025 Oct 3;487(5):959–69. doi: 10.1007/s00428-025-04260-w (PMC12647349; doi:10.1007/s00428-025-04260-w)
Supplement: Supplementary file 2 — Supplementary Material 1 (DOCX 36.7 KB) [file 428_2025_4260_MOESM2_ESM.docx]

**Supplementary Table 1**: Distribution of HCC cells within the three clusters

| **Case** | **Core** | **Cluster A** | **Negative Silhouette Score A** | **Cluster B** | **Negative Silhouette Score B** | **Cluster C** | **Negative Silhouette Score C** | **Main Cluster** | **Purity Score*** |
| --- | --- | --- | --- | --- | --- | --- | --- | --- | --- |
| 1 | 1 | 713 | 495 | 1326 | 9 | 31 | 1 | B | 0,826 |
|  | 2 | 378 | 316 | 335 | 1 | 41 | 2 | B |  |
| 2 | 1 | 153 | 66 | 1557 | 44 | 1 | 0 | B | 0,938 |
|  | 2 | 205 | 102 | 1376 | 1 | 0 | NA | B |  |
| 3 | 1 | 122 | 76 | 2445 | 79 | 512 | 50 | B | 0,854 |
|  | 2 | 1197 | 1067 | 1409 | 2 | 7 | 0 | B |  |
| 4 | 1 | 862 | 215 | 139 | 101 | 1019 | 37 | C | 0,553 |
|  | 2 | 1427 | 737 | 36 | 3 | 26 | 0 | A |  |
| 5 | 1 | 4881 | 150 | 108 | 49 | 48 | 1 | A | 0,987 |
|  | 2 | 3898 | 61 | 4 | 0 | 0 | NA | A |  |
| 6 | 1 | 2 | 0 | 584 | 0 | 6 | 6 | B | 0,997 |
| 7 | 1 | 0 | NA | 3 | 1 | 2689 | 10 | C | 0,996 |
|  | 2 | 36 | 23 | 4 | 0 | 2307 | 19 | C |  |
| 8 | 1 | 3 | 2 | 1937 | 155 | 1121 | 299 | B | 0,478 |
|  | 2 | 2406 | 1118 | 584 | 23 | 466 | 18 | A |  |
| 9 | 1 | 893 | 421 | 220 | 5 | 325 | 16 | A | 0,822 |
|  | 2 | 2829 | 203 | 152 | 9 | 6 | 0 | A |  |
| 10 | 1 | 1965 | 1108 | 27 | 26 | 1119 | 0 | C | 0,342 |
|  | 2 | 1724 | 1086 | 27 | 21 | 1754 | 0 | C |  |
| 11 | 1 | 155 | 62 | 429 | 296 | 7844 | 82 | C | 0,588 |
|  | 2 | 687 | 293 | 5438 | 112 | 784 | 69 | B |  |
| 12 | 1 | 48 | 24 | 1963 | 5 | 80 | 11 | B | 0,856 |
|  | 2 | 31 | 22 | 1027 | 1 | 436 | 38 | B |  |
| 13 | 1 | 2858 | 6 | 528 | 505 | 0 | NA | A | 0,950 |
|  | 2 | 2488 | 75 | 492 | 239 | 2 | 1 | A |  |
| 14 | 1 | 307 | 174 | 0 | NA | 207 | 1 | C | 0,608 |
| 15 | 1 | 108 | 91 | 116 | 115 | 1227 | 1 | C | 0,559 |
|  | 2 | 20 | 3 | 1608 | 5 | 8 | 5 | B |  |
| 16 | 1 | 965 | 182 | 1488 | 181 | 3 | 0 | B | 0,624 |
| 17 | 1 | 137 | 78 | 214 | 2 | 130 | 0 | B | 0,652 |
|  | 2 | 782 | 59 | 15 | 5 | 66 | 0 | A |  |
| 18 | 1 | 58 | 18 | 237 | 4 | 165 | 11 | B | 0,546 |
| 19 | 1 | 1578 | 391 | 122 | 18 | 144 | 20 | A | 0,839 |
| 20 | 1 | 1688 | 134 | 66 | 56 | 265 | 32 | A | 0,888 |
|  | 2 | 2424 | 203 | 337 | 156 | 55 | 2 | A |  |
| 21 | 1 | 69 | 9 | 3 | 2 | 12 | 0 | A | 0,822 |
| 22 | 1 | 2024 | 1164 | 538 | 44 | 442 | 1 | A | 0,479 |
|  | 2 | 1258 | 829 | 48 | 2 | 426 | 3 | C |  |
| 23 | 1 | 1004 | 17 | 0 | NA | 1 | 0 | A | 0,999 |
| 24 | 1 | 1770 | 190 | 69 | 25 | 0 | NA | A | 0,954 |
|  | 2 | 1903 | 182 | 226 | 111 | 0 | NA | A |  |
| 25 | 1 | 25 | 7 | 413 | 94 | 1587 | 55 | C | 0,570 |
|  | 2 | 197 | 181 | 1813 | 1 | 48 | 5 | B |  |
| 26 | 1 | 31 | 24 | 0 | NA | 2154 | 0 | C | 0,995 |
|  | 2 | 73 | 50 | 3 | 3 | 4220 | 0 | C |  |
| 27 | 1 | 1004 | 1 | 2 | 0 | 2 | 0 | A | 0,998 |
|  | 2 | 1642 | 3 | 0 | NA | 0 | NA | A |  |
| 28 | 1 | 530 | 174 | 1006 | 32 | 10 | 0 | B | 0,740 |
|  | 2 | 143 | 52 | 358 | 6 | 10 | 0 | B |  |
| 29 | 1 | 267 | 189 | 247 | 4 | 985 | 48 | C | 0,532 |
|  | 2 | 232 | 50 | 1211 | 22 | 115 | 54 | B |  |
| 30 | 1 | 15 | 11 | 4 | 0 | 1339 | 2 | C | 0,994 |
| 31 | 1 | 228 | 84 | 56 | 7 | 364 | 3 | C | 0,359 |
|  | 2 | 699 | 234 | 691 | 15 | 0 | NA | B |  |
| 32 | 1 | 98 | 21 | 85 | 10 | 8 | 0 | A | 0,646 |
|  | 2 | 1409 | 417 | 15 | 12 | 504 | 3 | A |  |
| 33 | 1 | 1 | 1 | 5 | 3 | 3342 | 1 | C | 0,629 |
|  | 2 | 806 | 635 | 2785 | 313 | 1150 | 15 | B |  |
| 34 | 1 | 765 | 252 | 277 | 174 | 1717 | 27 | C | 0,733 |
| 35 | 1 | 920 | 402 | 185 | 2 | 1281 | 36 | C | 0,518 |
|  | 2 | 831 | 266 | 464 | 11 | 639 | 37 | C |  |
| 36 | 1 | 3 | 2 | 0 | NA | 1240 | 0 | C | 0,998 |
|  | 2 | 1 | 1 | 8 | 1 | 2050 | 3 | C |  |
| 37 | 1 | 58 | 29 | 2683 | 0 | 0 | NA | B | 0,975 |
|  | 2 | 218 | 92 | 3320 | 6 | 2 | 1 | B |  |
| 38 | 1 | 218 | 64 | 31 | 14 | 171 | 0 | C | 0,510 |
|  | 2 | 770 | 246 | 32 | 18 | 450 | 1 | C |  |
| 39 | 1 | 1629 | 26 | 50 | 8 | 0 | NA | A | 0,974 |
| 40 | 1 | 751 | 413 | 1214 | 63 | 439 | 143 | B | 0,465 |
|  | 2 | 1663 | 202 | 701 | 79 | 3 | 0 | A |  |
| 41 | 1 | 650 | 414 | 2391 | 37 | 558 | 138 | B | 0,721 |
|  | 2 | 1750 | 848 | 2025 | 52 | 147 | 28 | B |  |
| 42 | 1 | 559 | 317 | 1073 | 0 | 0 | NA | B | 0,519 |
|  | 2 | 1429 | 258 | 242 | 8 | 0 | NA | A |  |
| 43 | 1 | 1356 | 734 | 278 | 0 | 28 | 2 | A | 0,792 |
|  | 2 | 708 | 22 | 41 | 2 | 0 | NA | A |  |
| 44 | 1 | 67 | 66 | 745 | 0 | 334 | 31 | B | 0,620 |
|  | 2 | 219 | 196 | 120 | 0 | 207 | 3 | C |  |
| 45 | 1 | 1226 | 39 | 22 | 1 | 26 | 0 | A | 0,962 |
| 46 | 1 | 0 | NA | 2110 | 0 | 67 | 66 | B | 0,999 |
|  | 2 | 0 | NA | 1862 | 0 | 232 | 231 | B |  |
| 47 | 1 | 673 | 416 | 521 | 16 | 4805 | 323 | C | 0,797 |
|  | 2 | 1065 | 695 | 900 | 45 | 3553 | 251 | C |  |
| 48 | 1 | 1049 | 502 | 503 | 7 | 6 | 0 | A | 0,521 |
| 49 | 1 | 960 | 101 | 0 | NA | 0 | NA | A | 1,000 |
| 50 | 1 | 262 | 113 | 499 | 10 | 6 | 0 | B | 0,795 |
|  | 2 | 78 | 20 | 579 | 11 | 174 | 114 | B |  |
| 51 | 1 | 7 | 3 | 27 | 20 | 3324 | 21 | C | 0,993 |
|  | 2 | 44 | 27 | 42 | 23 | 3128 | 31 | C |  |
| 52 | 1 | 11 | 10 | 18 | 7 | 2696 | 3 | C | 0,996 |
| 53 | 1 | 1208 | 523 | 14 | 0 | 72 | 1 | A | 0,916 |
|  | 2 | 1507 | 707 | 40 | 0 | 11 | 0 | A |  |
| 54 | 1 | 77 | 65 | 2013 | 0 | 0 | NA | B | 0,761 |
|  | 2 | 1135 | 287 | 741 | 8 | 2 | 0 | A |  |
| 55 | 1 | 1558 | 1044 | 6 | 0 | 14 | 0 | A | 0,963 |
| 56 | 1 | 2372 | 2175 | 800 | 2 | 600 | 28 | B | 0,489 |
|  | 2 | 3244 | 1733 | 199 | 6 | 234 | 15 | A |  |
| 57 | 1 | 542 | 391 | 598 | 0 | 4 | 0 | B | 0,794 |
| 58 | 1 | 1 | 0 | 4075 | 0 | 88 | 88 | B | 0,978 |
|  | 2 | 2 | 0 | 2578 | 7 | 925 | 777 | B |  |

*based on cells with positive Silhouette scores per case
